# Electronic Structure and Optical Property Analysis of Al/Ga-Codoped ZnO through First-Principles Calculations

**DOI:** 10.3390/ma9030164

**Published:** 2016-03-04

**Authors:** Chieh-Cheng Chen, Hsuan-Chung Wu

**Affiliations:** Department of Materials Engineering, Ming Chi University of Technology, New Taipei 24301, Taiwan; j29846892@gmail.com

**Keywords:** first-principles calculations, AGZO, electronic structure, optical properties

## Abstract

Using density functional theory and the Hubbard U method, we investigated the geometric structure, electronic structure, and optical property of Al/Ga-codoped ZnO. A 3 × 3 × 3 ZnO supercell was used to construct Al- and Ga-monodoped ZnO structures and Al/Ga-codoped ZnO (AGZO) structures. All three structures showed n-type conduction, and the optical band gaps were larger than that of pure ZnO. For a given impurity concentration, Ga impurities contribute more free carriers than Al impurities in AGZO. However, the presence of Al impurities improves the transmittance. These results can theoretically explain the factors that influence the electrical and optical properties.

## 1. Introduction

ZnO is an abundant, nontoxic material with a wide band gap (3.37 eV) and is transparent in visible light [1]. Recently, ZnO-based materials have been used in numerous photoelectric devices. However, the resistivity of pure ZnO is of the order of 10^−2^ Ω·cm, which is considerably greater than that of indium tin oxide (of the order of 10^−4^ Ω·cm). An effective method for improving the electrical properties of ZnO is doping with impurities such as Al, Ga, In, Co, and Mn [2]. Among these impurities, Al and Ga are the most widely used n-type dopants.

The Al dopant is easily oxidized during film growth because of its high reactivity, whereas the Ga dopant is more stable and resists oxidation [3]. In particular, the ionic and covalent radii of Ga are 0.62 and 1.26 Å, respectively, which are close to those of Zn (0.74 and 1.31 Å) [4]. Doping Ga in Al-doped ZnO (AZO) could reduce crystal deformation, resulting in higher crystallinity and enhanced electron transport [5]. Therefore, numerous researchers have successfully fabricated Al/Ga-codoped ZnO (AGZO) thin films and observed that these thin films show excellent electrical and optical properties [6]. Shin *et al.* [7] fabricated AGZO thin films with different Al/Ga concentrations by using pulsed laser deposition. They showed that doping 1.5 at % Al/Ga in AGZO thin films resulted in the lowest electrical resistivity of 2.18 × 10^−4^ Ω·cm and a transmittance of 85%. Liu *et al.* [8] demonstrated that AGZO thin films had superior electrical properties compared to AZO and Ga-doped ZnO (GZO) thin films and that the lowest resistivity of AGZO thin films was 8.12 × 10^−4^ Ω·cm. In addition, AGZO thin films have a higher average transmittance than the other two thin films in the wavelength range 300–1000 nm. Zhang *et al.* [9] observed that high Al or Ga doping concentrations reduced the number of oxygen vacancies in ZnO, resulting in a lower carrier concentration and higher resistivity. The theoretical calculations of AZO and GZO have been used to evaluate and explain the photoelectric properties of AZO and GZO. Bazzani *et al.* [10] proposed an explanation to the degradation of the optoelectronic properties of AZO at high Al doping concentration. The localized occupied states due to the presence of interstitial Al defects were flat bands, which act as traps for the optical transitions. Gabás *et al.* [11] indicated the decrease in the AZO film resistivity with Al doping is because of the filling of the Al impurity states, which pins the Fermi level just below the conduction band maximum. Palacios *et al.* [12] used density functional theory, including a Hubbard correlation term to correct the band gap problem, to study the resistivity of Al-doped ZnO with/without O vacancy structures. Their results showed that the Al donor states in the conduction band hybridize with the O-2*p* states, which decreases the resistivity of these structures. Our previous studies [13] have also adopted density functional theory and the Hubbard U method to explain the potential reasons for the shift in electrical and optical properties of Ga-doped ZnO structures under varying O flow rates.

As discussed, AGZO thin films obtained using various deposition tools have been widely studied and show excellent photoelectric properties. However, to the best of our knowledge, there is no theoretical report on the electronic structure and optical properties of AGZO. Theoretical calculations can provide information on materials at the microscopic scale. In this study, we used first-principles calculations to study the geometric structure, charge density, electronic structure, and optical properties of AGZO. The purpose of this study was to evaluate the roles of Al and Ga dopants in the AGZO structure. In terms of the design of doped structures, we constructed three structure (AZO, GZO, AGZO) at the same doping concentration in a 3 × 3 × 3 ZnO supercell, in which two Zn atoms were substituted with two dopant atoms. Therefore, the roles of Al and Ga atoms in the AGZO could be observed through comparing AGZO with GZO and AZO, respectively. Our results showed that the Ga atoms supply more free carriers than Al atoms in AGZO; the presence of Al increases the transmittance.

## 2. Calculation Models and Methods

**Figure 1:**
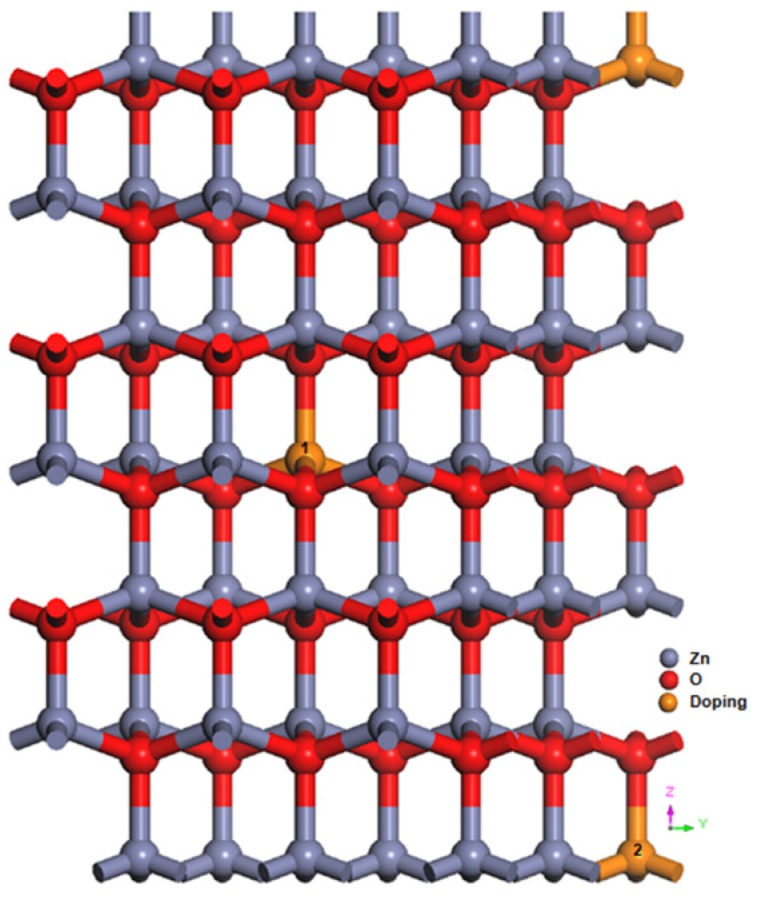
A 3 × 3 × 3 supercell model for Al/Ga-doped ZnO.

We used a 3 × 3 × 3 ZnO supercell with 108 atoms, and evaluated its geometric structure, charge density, electronic structure, and optical properties. In Figure 1, the red, gray, and orange spheres represent O, Zn, and doping atoms, respectively. On the basis of calculated values of the formation energy, Saniz *et al.* [14] found that the substitution of Al or Ga atoms at Zn sites yielded the most stable structure of AZO or GZO. Therefore, this study considered only AGZO structures in which Al/Ga atoms were substituted at Zn sites. To determine whether Al and Ga atoms tend to disperse or cluster in the AGZO structures, we calculated the total energies at three distances between Al and Ga atoms. The total energies of the far, medium, and near distances are −114,648.9, −114,648.893, and −114,648.793 eV, respectively. The total energy of the far distance is lower than those of the medium (0.007 eV) and near (0.107 eV) distances, indicating that the farthest distance corresponds to the lowest energy and that Al and Ga atoms tend to disperse. An AGZO model was constructed by substituting two Zn atoms (Sites 1 and 2 in Figure 1) with Al and Ga atoms. For comparison, two monodoped models, one each of AZO and GZO, were also constructed, in which two Zn atoms (Sites 1 and 2) were substituted with two Al or Ga atoms. The doping concentrations of these three models were approximately 3.7 at %.

In this study, all models were developed using the Cambridge Serial Total Energy Package [15]. Each model was structurally optimized before calculating its properties. Electron–ion interactions were modeled using ultrasoft pseudopotentials in the Vanderbilt form [15]. The valence configurations of the Zn, O, Al, and Ga atoms were 4*s*^2^3*d*^10^, 2*s*^2^2*p*^4^, 3*s*^2^3*p*^1^, and 4*s*^2^3*d*^10^4*p*^1^, respectively. A Monkhorst–Pack *k*-point grid of 3 × 3 × 2 was used [16], and a cutoff energy of 400 eV was considered for a plane wave. The parameters of the cutoff energy, and the Monkhorst–Pack grid were determined according to the convergence test. In the structural optimization process, the maximum displacement tolerance, maximum stress, maximum force, and energy change were set at 0.001 Å, 0.05 GPa, 0.03 eV/Å, and 10^−5^ eV/atom, respectively. The convergence threshold for self-consistent iterations was set at 10^−6^ eV. In our previous study [17], we adopted the density functional theory and the Hubbard U (DFT + U) method to successfully avoid underestimating the band gap. This study also used the DFT + U method to analyze the model properties [1].

## 3. Results and Discussion

### 3.1. Geometric Structure

Table 1 presents the lattice constants and average bond lengths after the structural optimization process. The optimized crystal parameters of pure ZnO were *a* = *b* = 3.281 Å and *c* = 5.296 Å, which are in accordance with experimental values of *a* = *b* = 3.250 Å and *c* = 5.207 Å [18]. For the AZO and GZO models, the Zn–O bonds were slightly longer than those in the pure ZnO model, and the Al–O (1.810) and Ga–O (1.907) bonds were shorter than the Zn–O bonds because Al^3+^ and Ga^3+^ ions with smaller radii (0.535 and 0.62 Å) replaced Zn ions, which had a larger ionic radius of 0.74 Å [19]. For the AGZO model, the Zn–O, Al–O, and Ga–O bond lengths did not show any apparent difference relative to the monodoped AZO and GZO models, and the bond length order was Al–O < Ga–O < Zn–O. In addition, the *c*-axis of the AZO model was the shortest among the *c*-axes of all three doped models. The ZnO (002) plane related to the ZnO *c*-axis, whereas the X-ray diffraction patterns ZnO (002) shifted to a higher or lower degree, which can be regarded as variations of the *c*-axis. In this study, when an Al atom in the AZO model was replaced with a Ga atom, the length of the *c*-axis increased from 5.301 to 5.306 Å, which is consistent with experimental results [20].

### 3.2. Charge Density

Table 2 presents the average Mulliken atomic and bond populations of the Al/Ga-doped ZnO models. Mulliken charges and bond populations are calculated in ONETEP according to Mulliken’s formalism [21]. The calculated Mulliken population could explain the charge transfer and bond type after bonding. A positive value for the atomic population indicates the degree of atoms losing an electron, whereas a negative value reflects the amount of atoms gaining an electron. In addition, a bond with a larger bond population has stronger covalent characteristics.

**Figure 2:**
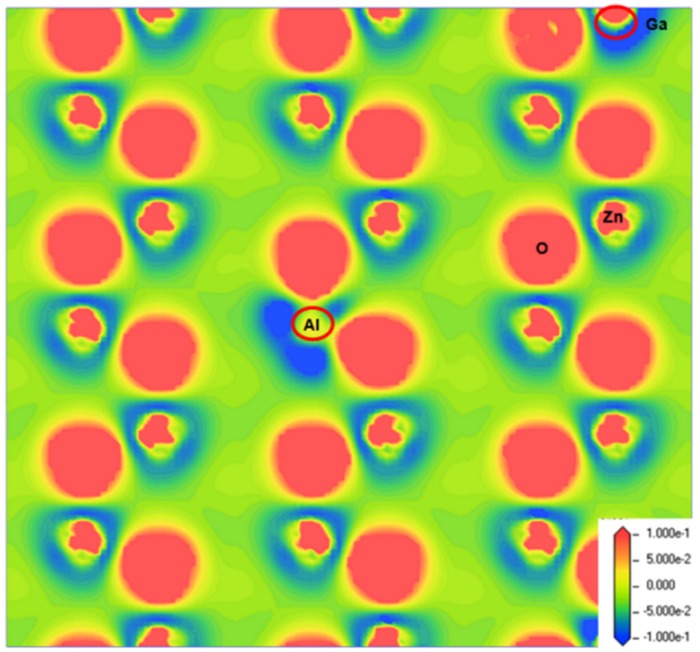
Distribution of charge density difference for the AGZO model.

Figure 2 presents contour plots of the difference in charge density in the AGZO model. To present the highest amount of atoms in the charge density difference, we chose the plane along the *Z*-axis in the AGZO model. For the pure ZnO model, the average population values of the Zn and O atoms were 0.94 and −0.94, suggesting that Zn atoms tend to lose electrons and O atoms tend to gain electrons. For the AZO and GZO models, the atomic populations of Al (1.61|e|) and Ga (1.37|e|) were larger than that of Zn (0.94|e|) because the valence of Al and Ga is higher than that of Zn. The atomic population of Al is larger than that of Ga, which should be the difference in electronegativity between Al (1.61) and Ga (1.81) [19]. For the AGZO model, the atomic populations of Al and Ga atoms were slightly smaller than those in the monodoped models, and the atomic population of Al was larger than that of Ga. This is consistent with Figure 2, in which more electrons tend to remain on a Ga atom (red color) compared to an Al atom. Furthermore, the bond populations of Al–O (0.498 |e|) and Ga–O (0.473 |e|) were larger than that of Zn–O (0.385 |e|), implying that Al–O and Ga–O bonds have stronger covalent characteristics than Zn–O has.

### 3.3. Electronic Structure

**Figure 3:**
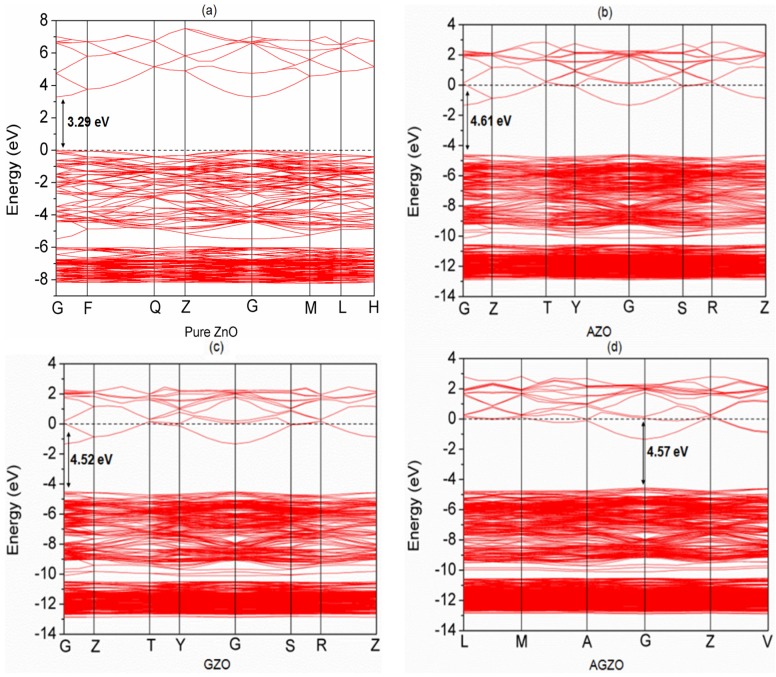
Band structures of (**a**) pure ZnO; (**b**) AZO; (**c**) GZO; and (**d**) AGZO.

Figure 3 shows the calculated band structures for pure and various Al/Ga-doped ZnO models. The Fermi level was set to zero and is represented by a dotted line. All the doped models (including the monodoped and codoped models) showed n-type conduction and shallow donor states at the bottom of the conduction band (CB), which is widely known as the Burstein–Moss effect [5]. Compared to the pure ZnO model, the AZO and GZO models showed larger optical band gaps of 4.61 and 4.52 eV, respectively. The optical band gap of the AZO model was larger than that of the GZO model, which is consistent with experimental results [8]. The optical band gap of the AGZO model was also larger than the band gap of the ZnO model [14], and its value was between those of the AZO and GZO models. One of the factors that affect the electronic mobility is the effective mass, which is related to the curvatures of the impurity band. Therefore, the band structures of Al/Ga-doped and codoped ZnO show that the curvatures of the impurity band are similar, meaning that the mobility of AZO, GZO, and AGZO are quite close.

**Figure 4:**
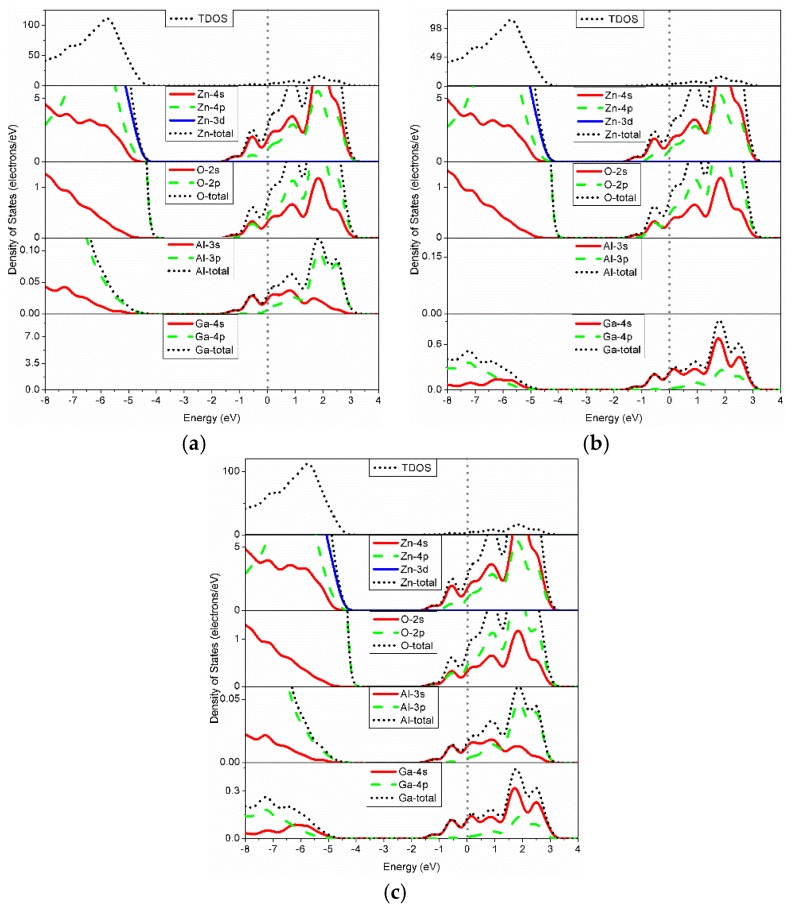
DOS of (**a**) AZO; (**b**) GZO; and (**c**) AGZO.

Figure 4 shows the density of states (DOS) for various Al/Ga-doped ZnO models. The CB mainly consists of s and p orbitals of Zn, O, Al, and Ga atoms, and the donor states are contributed by the s and p orbitals of the constituent Zn and O atoms and the Al-3*s* and Ga-4*s* states. To evaluate the contribution of each dopant atom to the carrier concentration, the shallow donor states for all atoms and each dopant were integrated. The calculated carrier concentrations are presented in Table 3. The total carrier concentration of the GZO model (1.753 × 10^21^ #/cm^3^) is higher than that of the AZO model (1.733 × 10^21^ #/cm^3^) for a given doping concentration. It can also be seen that Ga atoms contribute more free carriers than Al atoms. Experimentally, similar results have been obtained in other studies [8,22]. For the AGZO model, the carrier concentrations of Al, Ga, and all atoms are 8.557 × 10^18^, 7.505 × 10^19^, and 1.734 × 10^21^ #/cm^3^, respectively. Therefore, it can be concluded that Ga atoms contribute more free carriers than Al atoms in the monodoped and codoped models, which benefit conductive ability. In other words, more free carriers participate in the electrical transport process when Ga is incorporated in ZnO or AZO. It should be noted that the contribution of carrier concentration from an Al dopant in the AGZO structure is less than that from an Al dopant in the AZO structure; the contribution of carrier concentration from a Ga dopant in the AGZO structure is more than that from a Ga dopant in the GZO structure. In addition, that the electronegativity of Al is smaller than that of Ga is consistent with the calculated results of the atomic population (Al > Ga) mentioned in Section 3.2. However, Al atoms contribute less free carriers than Ga atoms. In Table 2 or Figure 2, the Al–O bonds have stronger covalent characteristics than does Ga–O. Therefore, this may be because more electrons lost from Al atoms participated to form covalent bonds.

### 3.4. Optical Properties

**Figure 5:**
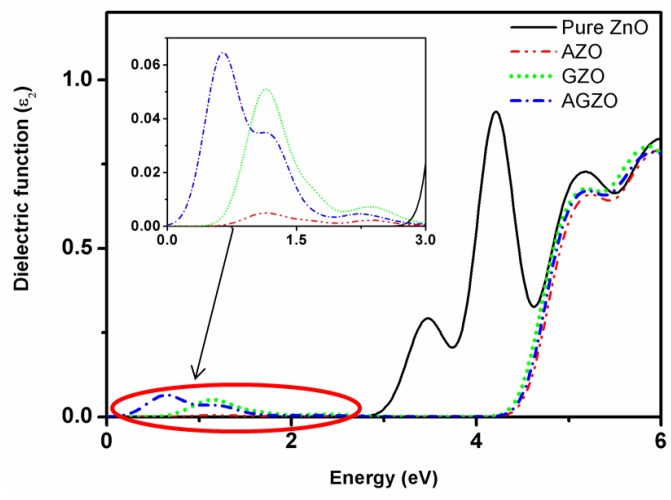
Imaginary part of the dielectric function of Al/Ga-doped ZnO.

The optical properties can be described using the dielectric function. Figure 5 shows the imaginary part ε_2_(ω) of the dielectric function and a magnified view of the Al/Ga-doped ZnO models. In this study, the transmittance was calculated from the absorption coefficient, reflection coefficient, and film thickness; the calculation has been explained in our previous paper [23]. We adopted polycrystalline to analyze the optical properties; no directions must be specified because the electric field vectors are adopted as a fully isotropic average. Moreover, the smearing is set at 0.2 eV in every model.

As shown in Figure 5, no absorption was observed for the pure ZnO model in the visible light region because of the wide band gap of 3.29 eV. For the AZO, GZO, and AGZO models, the peaks in the range from 0 to 2 eV were due primarily to the shallow donor state mentioned in Section 3.3. The shallow donor state results in increased absorption in the long-wavelength range of the visible light and infrared regions compared to the pure ZnO model. From the calculated band structure, the optical band gaps of the AZO, GZO, and AGZO models were 4.61, 4.52, and 4.57 eV, respectively. The large optical band gaps of these three doped models caused the blue shift of the absorption edge [7], resulting in a decrease in absorption in the visible light and ultraviolet (UV) regions compared to the pure ZnO model. In addition, the degree of blue shift (AZO > AGZO > GZO) followed the trend in the optical band gap.

Table 4 shows the average transmittance in the visible light region (400–800 nm) and UV region (200–400 nm). For the pure ZnO model, the transmittance was 88.4% in the visible light region and 64.9% in the UV region. For all doped models, the transmittances in both visible light and UV regions were higher than that of the pure ZnO model. In the visible light and UV regions, the AZO model showed the highest transmittance, followed by the AGZO and GZO models, in this order. Therefore, Al dopants could play a role in improving transmittance in AGZO model.

## 4. Conclusions

This study used the DFT + U method to investigate the electronic structure and optical properties of AGZO. The results showed that Al–O and Ga–O bonds were shorter than Zn–O bonds and showed stronger covalent characteristics. Doping Al, Ga, or both these elements in ZnO resulted in this compound exhibiting n-type conduction, larger band gap, and blue shift of the intrinsic absorption edge. For identical concentrations of Ga and Al dopants, Ga atoms supply more free carriers than Al atoms in AGZO; thus, the Ga atoms enhance the electrical conductivity. The average transmittance of AZO in the visible light and UV regions were 90.9% and 73.4%, respectively. These values were the highest among those of all the models constructed in this study. The presence of Al increases the transmittance.

## Figures and Tables

**Table 1:** Optimized lattice constants and bond lengths of Al/Ga-doped ZnO.

Structure	a (Å)	c (Å)	*c*/*a*	Bond Length (Å)		
				Zn–O	Al–O	Ga–O
Pure ZnO	3.281	5.296	1.614	2.002	–	–
AZO	3.277	5.301	1.618	2.009	1.810	–
GZO	3.284	5.312	1.618	2.009	–	1.908
AGZO	3.281	5.306	1.617	2.009	1.811	1.907

**Table 2:** Mulliken atomic and bond populations of Al/Ga-doped ZnO.

Structure	Atomic Population (│e│)				Bond Population		
	Zn	O	Al	Ga	Zn–O	Al–O	Ga–O
Pure ZnO	0.940	−0.940	–	–	0.400	–	–
AZO	0.921	−0.944	1.610	–	0.384	0.498	–
GZO	0.919	−0.932	–	1.370	0.386	–	0.475
AGZO	0.920	−0.938	1.600	1.360	0.385	0.498	0.473

**Table 3:** Calculated carrier concentration for Al/Ga-doped ZnO.

Carrier Concentration (#/cm^3^)			
Structure	PDOS-Al	PDOS-Ga	TDOS
AZO	1.779 × 10^19^	–	1.733 × 10^21^
GZO	–	1.347 × 10^20^	1.753 × 10^21^
AGZO	8.557 × 10^18^	7.505 × 10^19^	1.734 × 10^21^

**Table 4:** Average transmittance of Al/Ga-doped ZnO in the UV and visible light regions.

Structure	200–400 nm (%)	400–800 nm (%)
Pure	64.9	88.4
AZO	73.4	90.9
GZO	71.2	90.6
AGZO	72.8	90.8

## References

[1] Choi Y.J., Gong S.C., Johnson D.C., Golledge S., Yeom G.Y., Park H.H. (2013). Characteristics of the electromagnetic interference shielding effectiveness of Al-doped ZnO thin films deposited by atomic layer deposition. Appl. Surf. Sci..

[2] Li Z.Z., Chen Z.Z., Huang W., Chang S.H., Ma X.M. (2011). The transparence comparison of Ga- and Al-doped ZnO thin films. Appl. Surf. Sci..

[3] Shin S.W., Agawane G.L., Kim I.Y., Kwon Y.B., Jung I.O., Gang M.G., Moholkar A.V., Moon J.H., Kim J.H., Lee J.Y. (2012). Low temperature epitaxial growth and characterization of Ga-doped ZnO thin films on Al_2_O_3_ (0001) substrates prepared with different buffer layers. Appl. Surf. Sci..

[4] Chang S.C. (2014). In-line sputtered gallium and aluminum codoped zinc oxide films for organic solar cells. Int. J. Photoenergy.

[5] Ebrahimifard R., Golobostanfard M.R., Abdizadeh H. (2014). Sol-gel derived Al and Ga co-doped ZnO thin films: An optoelectronic study. Appl. Surf. Sci..

[6] Lee W., Shin S., Jung D.R., Kim J., Nahm C., Moon T., Park B. (2012). Investigation of electronic and optical properties in Al-Ga codoped ZnO thin films. Curr. Appl. Phys..

[7] Shin J.H., Shin D.K., Lee H.Y., Lee J.Y., Cho N.I., Lee S.J. (2009). Characteristics of gallium and aluminum co-doped ZnO (GAZO) transparent thin films deposited by using the PLD process. J. Korean Phys. Soc..

[8] Liu J., Zhang W.J., Song D.Y., Ma Q., Zhang L., Zhang H., Ma X.B., Song H.Y. (2014). Comparative study of the sintering process and thin film sputtering of AZO, GZO and AGZO ceramics targets. Ceram. Int..

[9] Zhang Z.Y., Bao C.G., Ma S.Q., Hou S.Z. (2011). Effect of crystallinity of ZnO buffer layer on the properties of epitaxial (ZnO:Al)/(ZnO:Ga) bi-layer films deposited on c-sapphire substrate. Appl. Surf. Sci..

[10] Bazzani M., Neroni A., Calzolari A., Catellani A. (2011). Optoelectronic properties of Al:ZnO: Critical dosage for an optimal transparent conductive oxide. Appl. Phys. Lett..

[11] Gabás M., Torelli P., Barrett N.T., Sacchi M., Bruneval F., Cui Y., Simonelli L., Díaz-Carrasco P., Barrado J.R.R. (2011). Direct observation of Al-doping-induced electronic states in the valence band and band gap of ZnO films. Phys. Rev. B Condens. Matter..

[12] Palacios P., Sánchez K., Wahnón K. (2009). Ab-initio valence band spectra of Al, In doped ZnO. Thin Solid Films.

[13] Lee M.H., Peng Y.C., Wu H.C. (2014). Effects of intrinsic defects on electronic structure and optical properties of Ga-doped ZnO. J. Alloys Compd..

[14] Saniz R., Xu Y., Matsubara M., Amini M.N., Dixit H., Lamoen D., Partoens B. (2013). A simplified approach to the band gap correction of defect formation energies: Al, Ga, and In-doped ZnO. J. Phys. Chem. Solids.

[15] Segall M.D., Lindan P.J.D., Probert M.J., Pickard C.J., Hasnip P.J., Clark S.J., Payne M.C. (2002). First-principles simulation: Ideas, illustrations and the CASTEP code. J. Phys. Condens. Mat..

[16] Monkhorst H.J., Pack J.D. (1976). Special points for Brillouin-zone integrations. Phys. Rev. B.

[17] Wu H.C., Peng Y.C., Chen C.C. (2013). Effects of Ga concentration on electronic and optical properties of Ga-doped ZnO from first principles calculations. Opt. Mater..

[18] Kisi E.H., Elcombe M.M. (1989). *u* parameters for the wurtzite structure of ZnS and ZnO using powder neutron diffraction. Acta Crystallogr. Sec. C.

[19] Macdonald F., Lide D.R. (2003). CRC handbook of chemistry and physics: From paper to web. Abstr. Pap. Am. Chem. S.

[20] Lin Y.C., Chen T.Y., Wang L.C., Lien S.Y. (2012). Comparison of AZO, GZO, and AGZO THIN FILMS TCOs applied for a-Si solar cells. J. Electrochem. Soc..

[21] Mulliken R.S. (1955). Electronic population analysis on LCAO–MO molecular wave functions. I. J. Chem. Phys..

[22] Shin J.H., Shin D.K., Lee H.Y., Lee J.Y. (2011). Properties of multilayer gallium and aluminum doped ZnO(GZO/AZO) transparent thin films deposited by pulsed laser deposition process. T. Nonferr. Metal. Soc..

[23] Wu H.C., Peng Y.C., Shen T.P. (2012). Electronic and optical properties of substitutional and interstitial Si-doped ZnO. Materials.

